# Straight roads into nowhere – obvious and not-so-obvious biological models for ferrophobic surfaces

**DOI:** 10.3762/bjnano.13.111

**Published:** 2022-11-17

**Authors:** Wilfried Konrad, Christoph Neinhuis, Anita Roth-Nebelsick

**Affiliations:** 1 Institute of Botany, Technical University Dresden, Zellescher Weg 20b, D-01217 Dresden, Germany; 2 Department of Geosciences, University of Tübingen, Schnarrenbergstr. 94–96, D-72076 Tübingen, Germany; 3 State Museum of Natural History Stuttgart, Rosenstein 1, D-70191 Stuttgart, Germany

**Keywords:** air-retaining interfaces, bioinspiration, biomimetics, biomimicry, blast furnace, Collembola, gas/liquid interfaces, interfacial effects, persistant air layers, pits, *Salvinia molesta*, surfaces, tuyère failure, water transport in plants, xylem, Young–Laplace equation

## Abstract

There are currently efforts to improve strategies for biomimetic approaches, to identify pitfalls and to provide recommendations for a successful biomimetic work flow. In this contribution, a case study of a concrete biomimetic project is described that started with a posed technical problem for which seemingly obvious biological models exist. The technical problem was to devise a ferrophobic surface that prevents the contact between the copper surface of a tuyère (a water cooled aeration pipe within a blast furnace) and liquid iron. Therefore, biological external surfaces that strongly repel liquids appeared to be suitable, particularly the hair cover of the water fern *Salvinia molesta* and the surface of Collembola (an arthropod group). It turned out, however, that it was not feasible to realise the functional structures of both biological models for the tuyère problem. Instead, a seemingly not obvious biological model was identified, namely micropores within the cell walls of water-transporting conduits of plants that connect the conduits to a three-dimensional flow network. These specially shaped pores are assumed to be able to create stable air bodies, which support the refilling of embolised conduits. By adopting the shape of these micropores, a successful prototype for a ferrophobic copper surface repelling liquid iron could be devised. This case study illustrates that straight road maps from technical problems to obvious biological models are no guarantee for success, and that it is difficult to arrive at a formalised biomimetic working scheme. Rather, a broad understanding of biological function and its complexity is beneficial.

## Introduction and Motivation

The basic concept of biomimetics is the derivation of technical applications from biological models. The expectation to find technically useful functional systems in living nature is commonly explained with evolution, because selection would lead to “smart” and “efficient” structures [1]. A huge pool of high-performance structures, practically “ready to harvest” would, therefore, be available. Engineers or other scientists from the applied sciences could benefit greatly from this natural resource by identifying biological “solutions to problems” and developing concepts of subsequent technical transfer.

Currently, two classes of biomimetic strategies are practiced. The first one, the “bottom-up” (or “biology push”) approach, denotes the introduction of identified biological functional systems into applied sciences, by directly suggesting possible technical applications. The second one, the “top-down” (or “technology pull”) approach, is initiated by a defined technical problem for which a suitable biological model is sought. The “Lotus effect” belongs to the first class. It was identified by Wilhelm Barthlott and suggested (later accompanied by one of the co-authors, C. Neinhuis) as relevant for surface technology. The highly water-repellent plant cuticles, which are equipped with hierarchically structured wax crystals, would represent “ready-made” models of superhydrophobic surfaces with versatile technical applications. The Lotus effect indeed proved to be attractive for applied sciences (as this special issue demonstrates), and its underlying physics was thoroughly studied.

Subsequently, more water-repellent biological structures were identified, with additional features and, therefore, higher and novel application potential [2–3]. For instance, various biological surfaces were described that are not only superhydrophobic but are additionally able to keep persistent air layers for an extended time after immersion in water. Principally, superhydrophobic surfaces are commonly surrounded by air when immersed. However, the air body is not persistent enough for most applications and dissolves after some time, in contrast to surfaces that can be described as “staying dry under water” for a much longer time [4–5]. Such surfaces usually feature functional structures that are larger than the wax crystals on the Lotus leaf (or other superhydrophobic leaf surfaces showing specially structured wax covers). Many of these surfaces possess hairs, such as those of the water spider or the floating fern *Salvinia molesta* (and other *Salvinia* species), and their surfaces have an appearance similar to that of terrycloth.

Both the Lotus effect and the surfaces with stay-dry-under-water potential became – after their introduction into biomimetics – popular items for the top-down strategy. They were included into various information resources on functional biological models, such as the popular and widely used “Ask nature” database (Biomimicry Institute, 2020. AskNature – Innovation Inspired by Nature. https://asknature.org/, [6]). In other words, once their functional potential is identified and described, biological systems migrate from the bottom-up realm to the top-down portfolio, together with their originally recognised application potential. Tapping this top-down portfolio is an attractive modus operandi because it appears to guarantee a straightforward and swift way to obtain the desired research results, apparently without any further need for biologists or biological expertise [7]. Another tool is TRIZ, a highly sophisticated approach that also focuses on a general biological design problem, namely trade-off conflicts [8–10].

The path from biological models to biomimetic prototypes or products can, however, be much less straightforward in practice. The complexity of the relationships between biology and technical applications leads to a high diversity of biomimetic projects with respect to procedures, strategies, project course and history [11–12]. Moreover, the biological model system and its functional aspects and contexts may not be fully understood and/or unraveled [1,7,13–14]. This hampers easy one-to-one translations of a (presumed) biological function to a technical solution or may even lead to potential conflicts with the originally intended technical application. Considering these difficulties, it appears to be desirable to obtain information on the state of the “biomimetic practice”. This is generally recognised, with various attempts to gain insight into how “biomimetic procedures” are conducted. As an example, one approach is to hand out assessment sheets asking concrete questions to involved scientists [15].

To obtain insight on factors that enhance or reduce the prospect of success, it can also be instructive to analyze the history and developments of biomimetic projects as case studies. In this contribution, we reconstruct the winding path of a project originally initiated by a technical problem that appeared to be solved straightforwardly by focusing on water-repellent biological surfaces (i.e., adopting a bottom-up strategy). As it turned out, however, the way to a finally successful prototype was not to follow the straight bottum-up road map. The project finally did not progress along a straight strategic road map. Instead, it developed within a “structure–effect space” shaped by biological complexities, challenging technical demands, initial failures and – importantly – results and insights from various previous projects. The actual project, whose history will be reconstructed in this contribution and in which two co-authors participated (C. N. and W. K.), dealt with a technical problem encountered during steel production. The aim was to find a (biomimetic) way to prevent the adhesion of liquid iron at 1500 °C to a water-cooled copper surface, an application whose technical demands are extremely far away from the conditions of organismic functions.

The aim of this contribution is to provide a case study from the biomimetic practice, representing an in-depth history of a biomimetic project. This history is represented by a sequence of projects in which the co-authors of this paper were involved in different combinations. The underlying biomimetic problems and physical as well as biological topics just provided the vehicle to describe the flow of concepts, inspirations and transfer problems.

## Perspective

### The problem: Failing blast furnace tuyères

**Figure 1:**
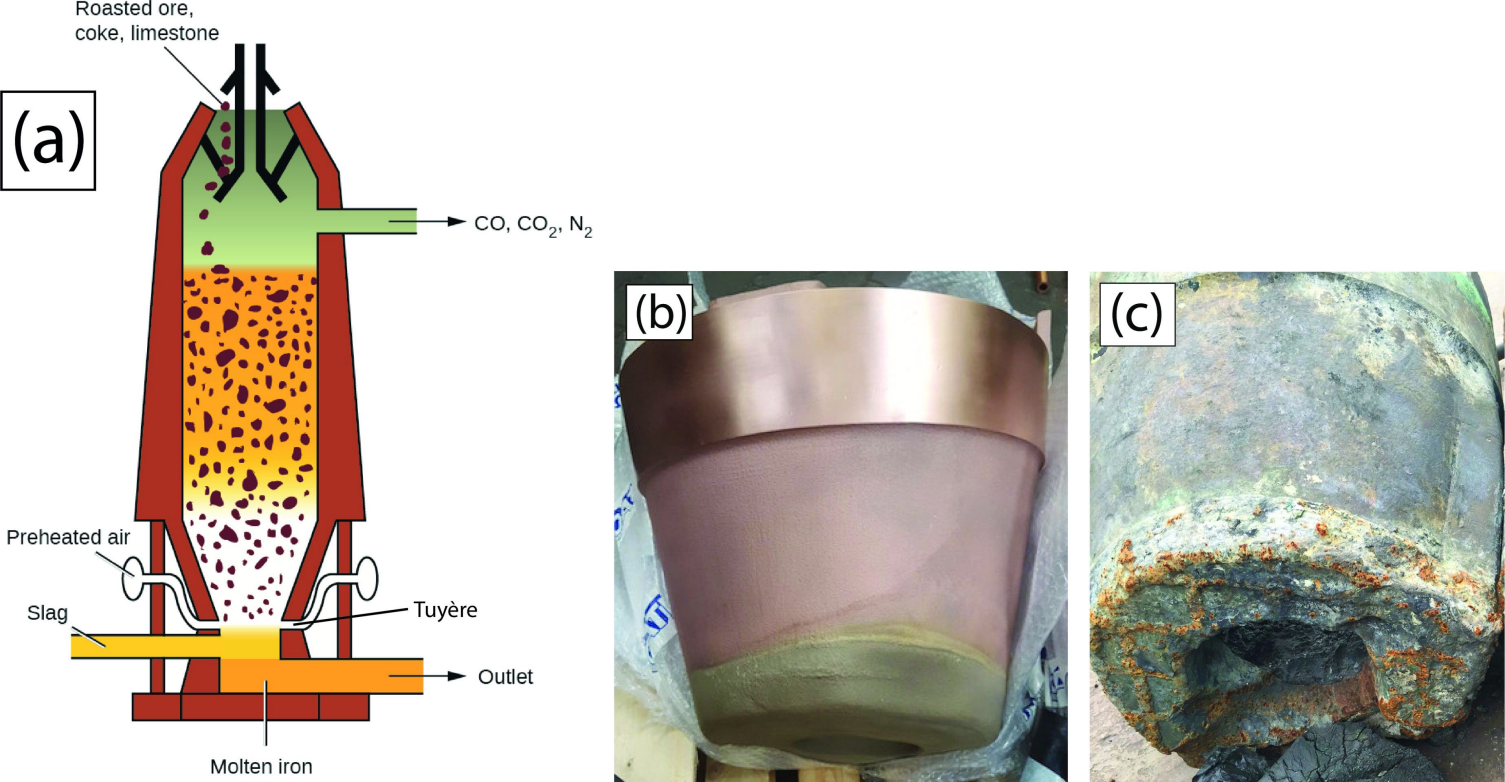
Left: Scheme of a typical blast furnace (picture from OpenStax, Blast Furnace Reactions, CC BY 4.0). Centre, right: a novel tuyère and a tuyère after a few weeks of usage; the lower parts extend into the blast furnace. (Figure 1a was reproduced from [16]. Accessible for free at https://openstax.org/books/chemistry-2e/pages/1-introduction (© 2019 P. Flowers et al., published by Openstax, distributed under the terms of the Creative Commons Attribution 4.0 International License, https://creativecommons.org/licenses/by/4.0). Figures Figure 1b,c: Photos by Siegfried Konietzko. This content is not subject to CC BY 4.0)

In this section, the problem and its technical and economical relevance will be briefly outlined. Iron ore consists mostly of iron oxides, which have to be reduced in order to obtain metallic iron (or steel). This is primarily achieved via the blast furnace pathway: iron ore, coke and huge amounts of very hot air are supplied to a blast furnace (Figure 1a). Coke and air produce hot carbon monoxide, which reduces the iron ore to liquid metallic iron. The hot air is pressed into the lower part of the furnace via double-walled, water-cooled pipes called tuyères that extend a short distance into the blast furnace (Figure 1b,c).

Occasionally, liquid iron that runs down along the inner side of the outer wall of the furnace makes contact with the tuyère. If the heat input exceeds the capacity of the tuyère’s water cooling system, the tuyère burns through, and, as a result, (liquid) water is injected into the oven. This requires the immediate deactivation and exchange of the tuyère because abruptly evaporating water may cause major destruction in and around the furnace.

Tuyère failure entails the shutdown of the blast furnace (in fact, it is the main reason for shutdowns [17–20]) and of the facilities for subsequent processing of iron and steel. This results in huge energy losses and economic cost due to additional heating and idle running of the periphery of the blast furnace. To find prospects to reduce such losses, the Betriebsforschungsinstitut at Düsseldorf (a research institute of the German steel industry) formed a collaboration with Hundt & Weber at Siegen (a manufacturer of tuyères) and the Institute of Botany of the Technical University of Dresden (C. N. and W. K.). The collaboration formulated the project proposal “Increase of energy efficiency of blast furnaces by using novel longlife-tuyères”, which was approved by the German Federal Ministry of Economy and Energy and lasted from February 2015 to July 2018.

The concept was to equip the tuyère surface with a structure that mitigates heat input into the tuyère, in order to relieve the tuyère’s water cooling system and to prevent heat damage to the tuyère. Since the heat conductivity of gas layers is about five orders of magnitude lower than that of copper, gas layers trapped in structured tuyère surfaces should substantially reduce the overall heat flow into the tuyère, and its outer walls should be much less prone to melting. The central idea was, therefore, to create a surface on the tuyère able to maintain an air layer that isolates the tuyère from the hot iron. For the persistence of the air layer, the following necessary conditions were identified:

- The surface should create a gas/liquid interface and the forces acting on this interface should be in stable mechanical equilibrium, that is, the (extra) forces generated by a (small) displacement of the interface should be able to push the system back to its equilibrium position.
- In case of liquid iron flowing over the tuyère, persistence of the gas pockets within the structured surface should be energetically favourable over their displacement.
- The interfaces that separate the gas pockets from the liquid iron should form autonomously, that is, without the need for external manipulations.
- The typical dimensions of the structures creating the air layer should not exceed the capillary length, that is, surface forces should dominate forces trying to distort the gas/liquid interface (e.g., gravitational forces).

### The straight top-down pathway: Identifying and following obvious biological models

**Figure 2:**
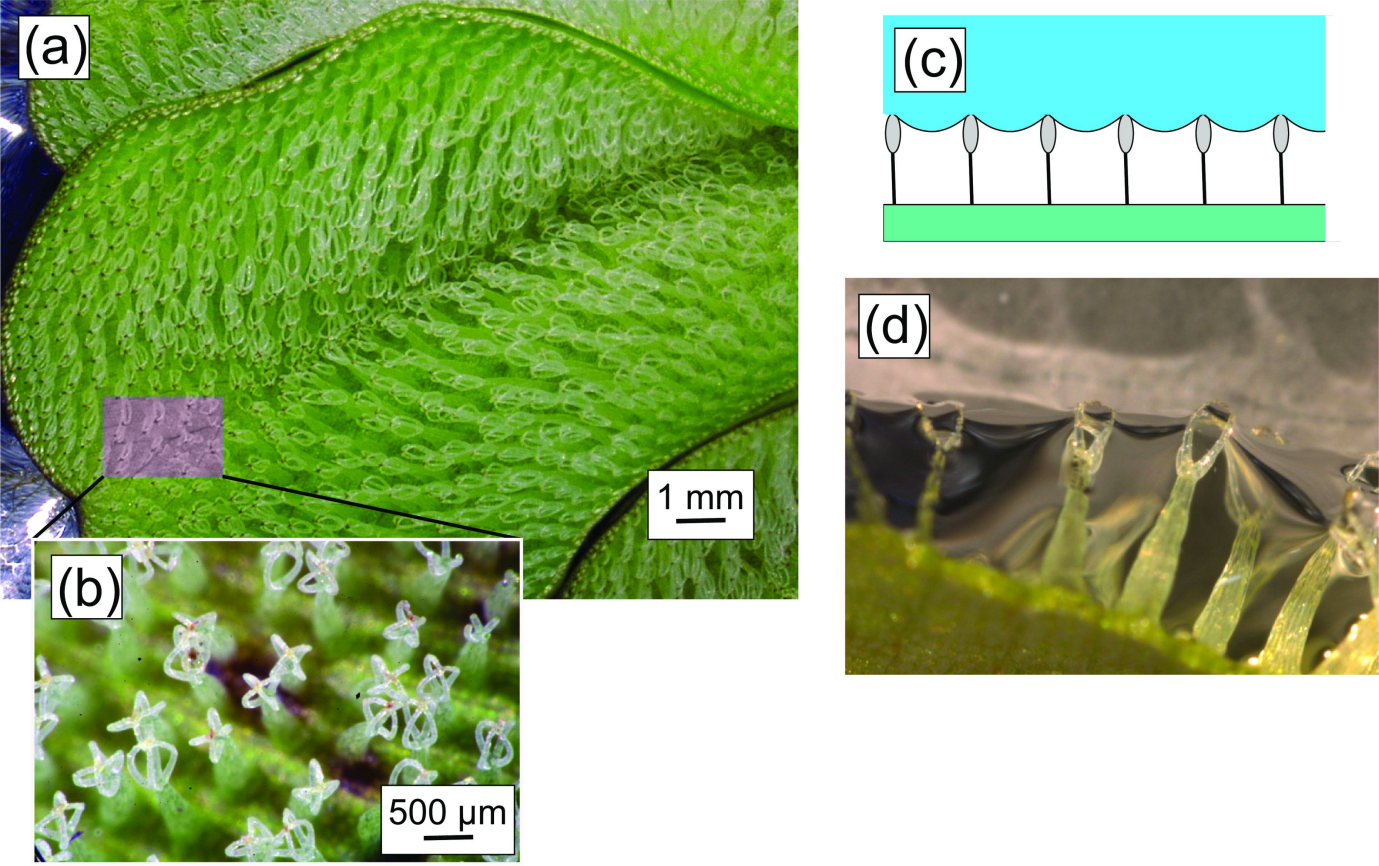
(a) A leaf of *Salvinia molesta* (Kariba weed) from above. The inset (b) shows the eggbeater-like structures. (c, d) Cross sections (schematic and photograph) through a leaf showing the pillar-like surface structures that seem to support the air/water interface covering the air layer.

**Figure 3:**
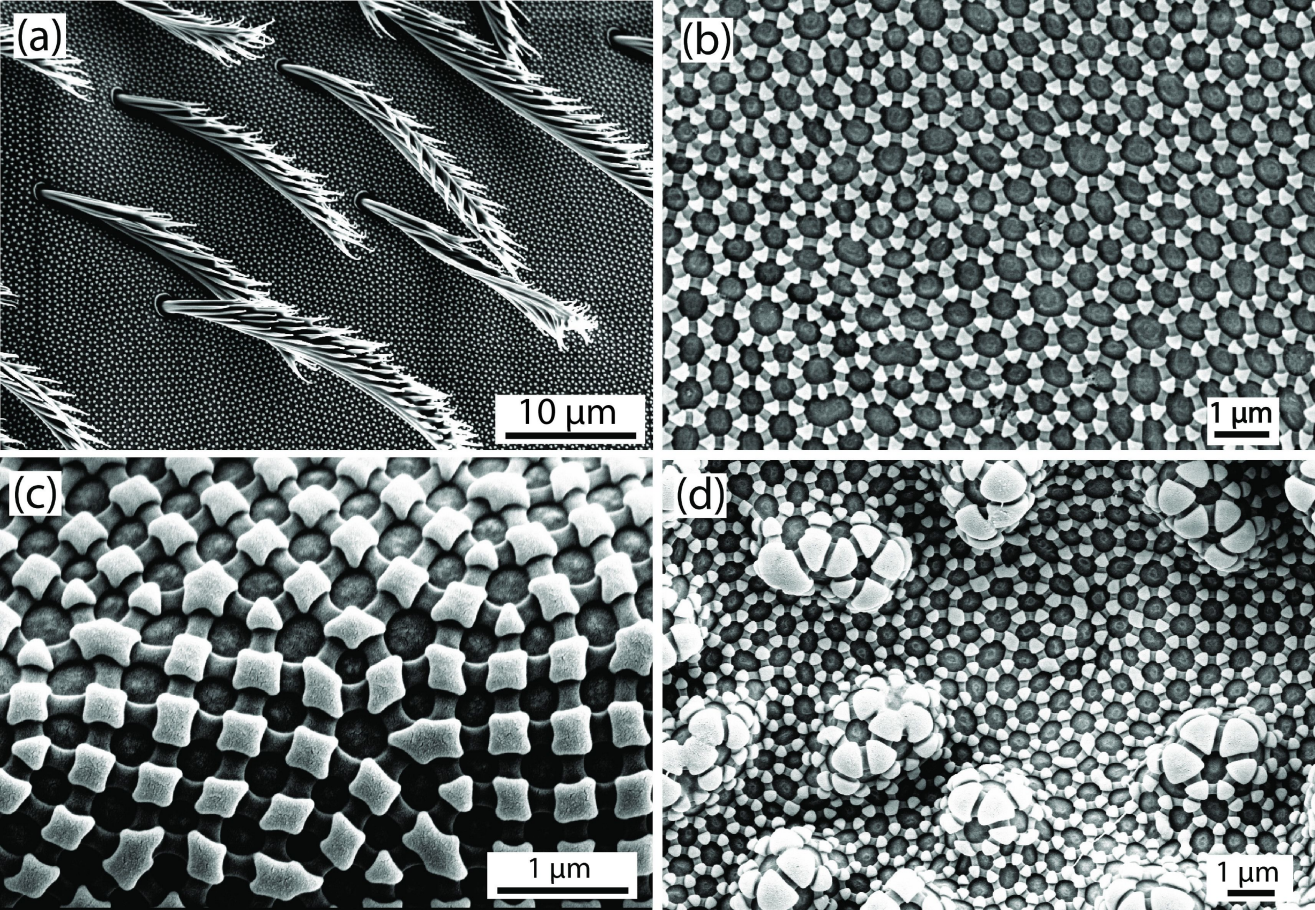
The skin structures of Collembola (springtails) show several levels of protection against wetting: (a) a hairy cover of bristles of *Sinella tenebricosa*, (b) nanoscopic hexagonal or rhombic comb structures formed by interconnected ridges in which gas bubbles can be trapped (*Ceratophysella scotica*) and (c) still smaller, very special structural elements with overhanging topographies (“undercuts”) at each corner of the combs (*Ceratophysella scotica*). (d) Depending on the species, secondary granules may be present, which constitute an additional layer of structuring between the bristles and the combs (*Folsomia candida*). Pictures were taken from the same specimens as in [23].

As a first step, it appeared to be quite straightforward to search for suitable biological models of external surfaces that prevent the contact to a liquid and that are able to create stable air/liquid interfaces resilient to perturbation. The biological models we considered first were the structured surfaces of *Salvinia molesta* leaves (see Figure 2) and the skin structure of springtails (Collembola, see Figure 3) [3,21–22], both of which are known to accommodate gas layers.

#### Kariba weed (*Salvinia molesta*)

In the case of *Salvinia molesta*, stable water/air interfaces form at the tips of leaf hairs, which are topped by eggbeater-like structures (Figure 2). These are able to hold the resulting air layer for extended periods of time upon immersion. The interface is also resilient against perturbations. The “*Salvinia* effect” has considerable biomimetic potential, for instance, with respect to fuel reduction in maritime shipping [24–25] because an air layer between ship hull and water reduces the drag considerably.

The physical basis of the capability of the *Salvinia* hairs to hold persistent air layers was the topic of various studies [5,26], which also included their elastic properties [27–28]. Main factors for the persistence and resilience of the air/water interface are the peculiar combination of the superhydrophobic surface of the hairs with the hydrophilic tip of the “eggbeater” structure [5] and the circumstance that, upon perturbation, for example, upon pressure changes, they allow for an “air spring” effect within the trapped air [26]. The *Salvinia* hair type appears to represent the result of evolutionary adaptation to a high performance and is, therefore, considered as an attractive biological model for applications requiring surfaces that remain dry upon immersion [5,29].

#### Springtails (Collembola)

Springtails (Collembola, Figure 3) are a group of small hexapods that occur worldwide in virtually all habitats. The number of described species approaches 10,000 [30] but is estimated to exceed 50,000 [31–32]. The name springtails originates from a tail-like appendage, the furca. It allows most of the species to jump in backflip style across distances of up to 10 cm. This is remarkable in view of their size, which rarely exceeds 1 mm, with a few exceptions of about 6 mm.

Most Collembola species are soil-dwelling organisms, which live in leaf litter, on the soil surface or in small spaces between soil particles. They feed on a variety of food sources and contribute substantially to the recycling of organic matter [32–33]. Collembola breathe through their body surface via skin breathing as well as with trachea [34]. Therefore, water films that may form as continuous layers on their body pose the risk of reducing respiration. As small hexapods, Collembola are furthermore at the risk of drowning during rain or when displaced into water bodies. To avoid suffocation and to secure oxygen supply under such conditions, Collembola form air layers on the body surface, so called plastrons, which are established above the skin via hierarchically organised hydrophobic surface structures when forced under water [3,21–22]. There are three levels of protection against wetting in Collembola: (i) a hairy cover consisting of bristles with water pinned to the tips (Figure 3a), (ii) nanoscopic hexagonal or rhombic comb structures formed by interconnected ridges in which gas bubbles can be trapped (Figure 3b) and (iii) still smaller, very special structural elements with overhanging topographies (“undercuts”) called primary granules that are found at each corner of the combs (Figure 3c). Depending on the species, secondary granules may be present, which constitute an additional layer of structuring between the bristles and the combs (Figure 3d). Depending on systematic group and habitat, the surface structure is highly variable without changes of the basic pattern [21,23].

These specific surface structures are remarkable in so far as air layers are observed to form in liquids of diverse chemical compositions, including organic solvents and oils, which show a wide variety of surface tension values and contact angles with respect to the skin [3,21]. This “omniphobic” property allows the Collembola to live in areas of soil or water bodies that contain a high concentration of organic liquids that are, for instance, the product of decay processes and may reduce considerably the surface tension of water. In addition, the plastron is able to withstand external pressure exceeding 0.4 MPa. The air, which is not able to escape from the pockets formed by the honeycombs, is compressed but expands again as soon as the pressure is released, reestablishing the plastron [21–22].

#### The straight pathway does not provide a solution

**Transfer problems with the biological models.** As fascinating as the air-holding hair cover of *Salvinia* and the surface structures of Collembola are, and despite the circumstance that these structures appear to fulfill all necessary conditions, the functional principles of these biological models are based on various properties that are not easily reproduced technically, at least not for the considered problem. With respect to the *Salvinia* hair, the combination of hydrophilic tip and superhydrophobic rest is difficult to realise, and particularly so for the current problem of protecting a metal body from hot iron. The working principle of *Salvinia* is based on a structural complexity that exceeds the technical feasibility (apart from the economic cost/benefit ratio, which is always a kind of elephant in the room for applied projects). Also, the surface elements of Collembola, particularly the undercut structure, are complex and difficult to realise for the pursued project.

Therefore, a simpler structure was sought, such as rigid pillars. In fact, various studies addressed the properties and application potential of this kind of technical surface structuring, for superhydrophobic surfaces as well as for surfaces with persistent air layers. Therefore, interfacial effects at surfaces covered with rigid columns have been well investigated phenomenologically [26,35–37]. There exists, also, a theoretical study from a former project, which will be shortly described in the following section.

**Experience from previous studies.** In a previous project, carried out by two co-authors of this study (W. K. and A. R.-N., “Drag-reducing air–water interfaces”, see appendix A), it was attempted to tackle the problem of water/air interfaces at pillared surfaces theoretically. Principally, such an interface can be described by a differential equation. The main obstacle, however, is the complexity of this differential equation whose solutions describe – for given boundary conditions – the interface: It is non-linear and can only be solved if the interface (and its columnar supports) exhibit a high degree of symmetry (e.g., rotational symmetry, see below and appendix B, Equation 1 and Equation 2). Therefore, it is restricted to simple pillar shapes. Also, the arrangement of the pillars is essential. In a real *Salvinia* leaf, both quadratic or hexagonal patterns of hair arrangement may be recognised. This is unfortunately not sufficient to allow for a closed solution of the differential equation.

Konrad et al. [38] and Apeltauer [39] tried to circumvent this problem by establishing the existence of solutions and the stability and persistence of the related interface not from the solution but from properties of the differential equation itself. Their approach worked for hexagonally or quadratically arranged (Figure 2) circular columns of constant diameter and constant contact angle, but proved impracticable for more realistic protrusion models showing eggbeaters with hydrophilic tips (implying non-constant column diameter and contact angle, see the project “Drag-reducing air–water interfaces” in appendix A).

Approximating the eggbeater-like protrusions, however, by circular columns of constant diameter and constant contact angle, Konrad et al. [38] and Apeltauer [39] arrived at an interesting result: If the columns are hexagonally arranged, an interface trapping an air layer exists for arbitrary values of (i) the contact angle, (ii) the diameters of the columns and (iii) the distances between the columns. In case they are quadratically arranged, however, certain contact angle/size combinations have to be excluded because the related interfaces cannot exist. This result illustrates how seemingly simple properties are crucial for the persistence of the liquid/water interface and that the underlying physics is too complex for making straightforward assumptions and predictions about successful surface designs. Despite – or perhaps because of – the problems of the theoretical approach, more experimentally oriented research projects have been realised with the objective to fabricate drag-reducing surfaces for ship hulls [25].

**Leaving the straight pathway.** Therefore, no comprehensive “blueprint” from which such a simply surfaced structure could be devised exists so far, and, depending on conditions and application, the interfaces at a pillared surface may not be stable. As a consequence, there is no guarantee that an extensive empirical investigation of various test objects will be successful in identifying a suitable surface. To summarise, the interaction between *S. molesta* or Collembola and water does not constitute a good model for the interaction between liquid iron and blast furnace tuyères because of the following considerations:

- As described above, existing mathematical models are highly idealised and cover only partial aspects of the interaction between plant or animal, water and air layer. The identification of a suitable surface requires, therefore, empirical studies. The standard engineering approach to close such gaps of knowledge is to evaluate a series of experiments in which the “system-defining parameters” are systematically varied. In a blast furnace, however, where temperatures between (roughly) 1000 and 2500 °C prevail, it is, at best, possible to continuously measure the temperature of a tuyère, but no further experimental studies are possible within such an extreme environment. This means that in the considered project, such kind of systematic investigation was not possible.
- Furthermore, putting such an “experimental approach” into practice would mean to repeatedly replace differently designed tuyères, requiring each time a partial shutdown of the furnace. Since even a partial shutdown of a blast furnace is (economically) very expensive, operators are extremely reluctant to make concessions to scientists who would like to perform experiments. (Ideally, blast furnaces produce without interruption. Only after 10 or 20 years major renovations are to be expected). This means that in the considered project such kind of systematic investigation would have been too costly.
- A blast furnace holds several thousand tons of iron ore and coke, which are supposed to subside continuously downwards, following gravity. But sometimes cavities do form that entail avalanches of the ore and coke conglomerate within the furnace. Since a tuyère protrudes a short distance into the furnace, its surface is exposed to abrading encounters with the processed material. It is expected that delicate protruding structures devised according to the biological model systems of *Salvinia* or Collembola (see Figure 2 and Figure 3) would suffer from erosion.

It should be emphasised that the problem lies, therefore, less in the transfer of a system consisting of biological tissues and water working at room temperature to an arrangement of a metallic device and hot liquid iron. Rather, (i) the structure of the original biological models is too complex for an economically realistic solution, (ii) the derived structures are too fragile for the application, (iii) when attempting to fall back on to a simpler solution, the interfacial physics is too complex to identify a functional construction and (iv) empirical experimental studies are not possible in the considered project, for practical and economic reasons.

### Identification of a not-so-obvious biological model

#### An improbable biological model for ferrophobic surfaces: xylem structures of vascular plants

In a former biomimetic project (the research project “Hydrotex”, see appendix A), in which one co-author (W. K.) participated, the project goal was to construct a drag-reducing surface for boat hulls with textile materials, including tests of a prototype. In this case, the biological model of *Salvinia*-like protrusions was also initially in the focus, following a top-down process. In the course of the project, after experiencing some difficulties with the development of stable air layers on the *Salvinia*-like textile structures, another strategy was pursued based not on protrusions, but on the opposite, namely indentations, which in fact provided promising results (see appendix A).

The origin of the idea to use recesses resembling cones as air-holding structures can be traced back to another research project already mentioned in an earlier section (“Drag-reducing air–water interfaces”, see appendix A). The concept of a surface furnished with indentations instead of protrusions was also based on a biological model, although with a completely different functional background, which has, at first sight, nothing to do with water-repellent biological surfaces. It originated from research dealing with the xylem, the water transport system of plants, which had been pursued by two-co-authors for many years (A. R.-N. and W. K.). A technical exploitation of the transport principle was aimed at in the research project “Energy self-sufficient fibre based long-distance transport of liquids” (see appendix A).

The xylem is the plant tissue that transports water from the roots to the leaves. In trees and shrubs, the xylem is represented by the wood. It consists of conduits of dead cells forming capillaries in the lumina of which the water flows. To understand how this tissue can be the source for a biomimetic concept of a ferrophobic surface, some principles and problems of water transport in plants have to be briefly summarised.

The water transport system of vascular plants has to lift water against gravity from the roots to the leaves where it evaporates. The evaporation at the leaves (i.e., transpiration) represents the driving force for the flow, meaning that the water is not “pumped” upwards but rather “sucked”. Plant water transport relies, therefore, on two basic principles [40]: (i) transpiration, occurring in the leaves, providing the driving force for the water flow to overcome gravity and (ii) the cohesion of water, provided by van der Waals forces between the water molecules.

**Figure 4:**
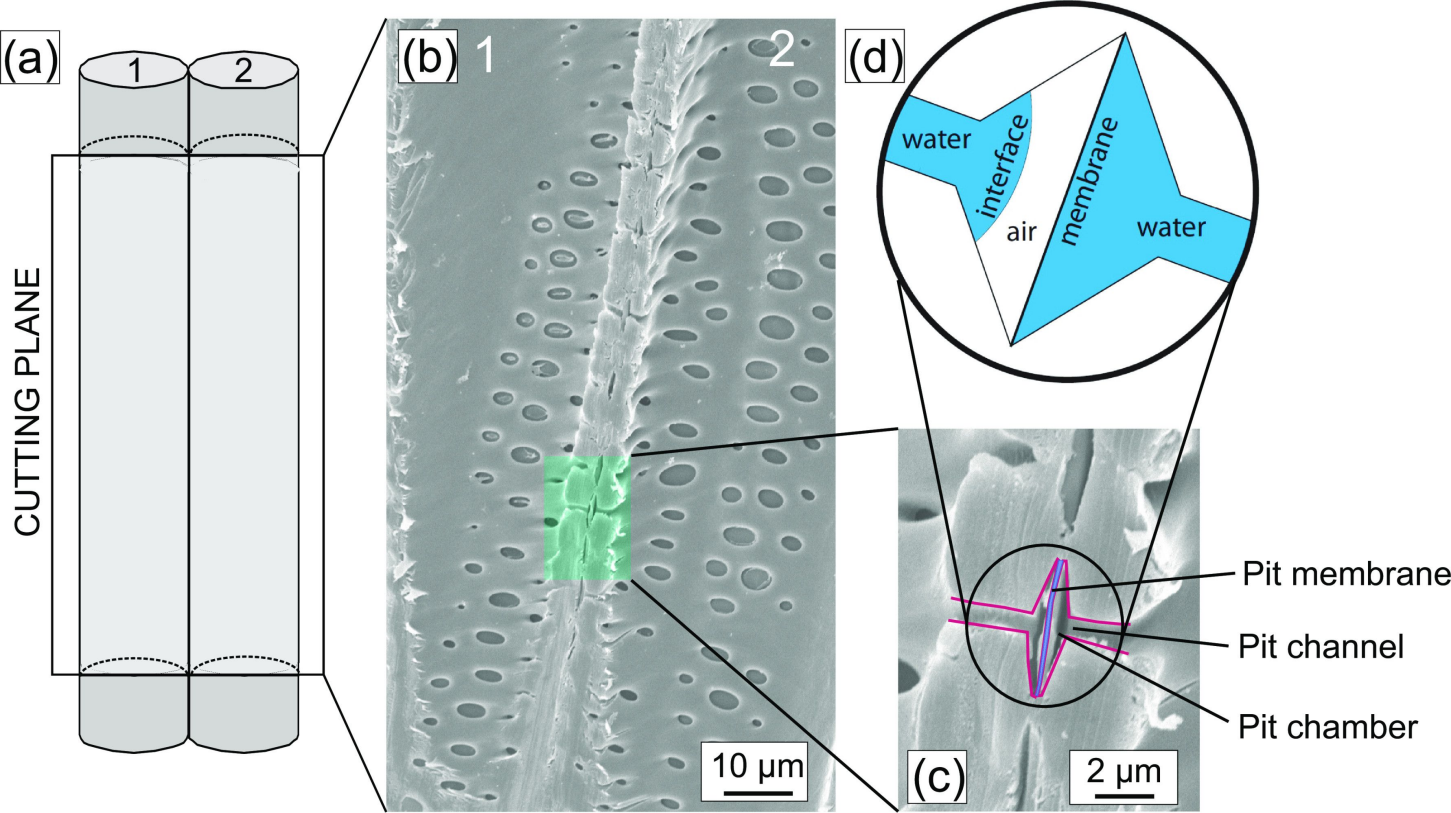
Basic structure of the xylem, the water transport tissue of plants. The xylem consists of elongated conduits, which are arranged parallel to the long axis of the plant stem, and which are connected to each other by pores termed “pits”. Panel (a) shows a sketch of two adjacent conduits cut open longitudinally. Panel (b) shows a scanning electron microscope image of two adjacent conduits (denoted “1” and “2”). The conduits are connected by pits (some are highlighted by the green area) containing the pit membrane, which is a special nanoporous membrane. (c) Detailed image showing a pit pore connecting the two conduits. The pore resembles two funnels (highlighted by a red outline) that are attached at their wide ends forming the pit chamber. The narrow ends form the pit channels. The pit membrane separating the two funnels is highlighted in blue. (d) During repair (after one of the conduits has become dysfunctional due to gas filling), the refilling conduit is separated from its still functional water-filled neighbour by an air pocket. This air space is stable due to the interfacial forces created by the shape of the pit chamber.

The conduits consist of the cell walls of the dead xylem cells. The water molecules flowing inside them are connected to each other and to the conduit walls by cohesion generated by van der Waals forces. The conduits are interconnected by pores, termed “pits”, which allow water to enter and leave the conduits on the way upwards (see Figure 4). In this way, a three-dimensional network flow of water molecules is formed whose upper end is fastened within the leaves where transpiration takes place at the living leaf cells. From there it “hangs down” through twigs, trunks, stem and roots into the ground water.

The stability of this transport mechanism relies on the comparatively weak van der Waals forces, whose range is at most 100 nm [41]. It is, therefore, prone to become unstable upon perturbations, particularly upon the entry of small air bubbles (by, for instance, damage such as broken twigs). If large enough, these perturbations initiate a process termed “air seeding”, which disrupts the water chain and causes conduit failure by the development of embolism, meaning gas (air) spaces interrupting parts of the transport pathway [42].

Because air seeding events are inevitable, trees developed safety measures to minimise the consequences. Of essential importance is the segmentation of the water conduit network into compartments. Each pit pore connects two conduits, and this joint pore space is separated by a special nanoporous membrane, the pit membrane (Figure 4). When a conduit becomes dysfunctional due to embolism, the nanopores within the pit membranes prevent the spreading of air into conduits that are connected to the dysfunctional conduit. Thus, the embolised conduit becomes hydraulically isolated from the functional ones (Figure 4).

Embolised conduits may be repaired, meaning that they become “refilled” again with xylem water. This is supposed to happen when transpiration ceases and the “suction force” within the conduits reduces. The reason is straightforward: An incompletely repaired conduit would not fill with water but would be sucked empty again and again because the pressure in the adjacent conduits is lower than in the still dysfunctional conduit. Therefore, a mechanism was suggested that would be able to isolate a refilling conduit during the repair process [43].

An essential element of this special isolation mechanism is the formation of a stable interface (denoted “interface” in Figure 4e), which separates water and air within the pit cavity, thereby isolating the pit membrane from water (see appendix C). Various research was dedicated to this putative mechanism. Studies contributed by Konrad and Roth-Nebelsick [44–45], who considered the interfacial physics of the proposed mechanism, provided evidence that such an interface may be possible within pits. Its stability would depend on the water pressure, shape of the pit and the contact angle between water and pit walls (see appendix C and [45]). Central to the formation of an interface is the shape of a pit. It resembles two funnels connected at their wide ends with the narrow ends (the pit channels, Figure 4) opening into the two adjacent conduits.

#### The working principle of the “ice cream cone” derived from xylem pits

At first sight, the funnel-like pit structures and the working principles governing the interface behaviour within the pit lumen appear to have nothing to do with persistent gas layers around immersed objects, such as ship hulls or blast furnace tuyères. However, the proposed working principle, which was originally suggested to explain the possible refilling of embolised conduits despite adverse pressure conditions, means nothing else than the persistence of air spaces between a solid and a liquid.

A closer look at the funnel structure (Figure 4) reveals that only a part of it is required for the creation of a stable interface. The narrow channel of a pit funnel, the pit channel, is just the connection to the conduit interior. Only the wider part of the funnel, the pit chamber (Figure 4), is functional with respect to interface formation. Principally, the upper funnel part can be closed at its bottom by elongation of the tilted funnel walls. The result is a simple cone with a special but highly symmetric shape. In applications, this structure would not protrude from a surface but would rather be embedded, forming robust recesses for creating stable interfaces to maintain persistent air spaces and prevent them from being pushed away by perturbations.

In contrast to covering surfaces with pillars or other protrusions, the pit-based principle is based on cavities, which are much more protected against abrasion and erosion than pillars or columnar structures. Furthermore, surface structures such as the pillars of *Salvinia* (Figure 2d) or the discontinuous hexagonal ridges of *Ceratophysella scotica* (Figure 3b) will probably generate a joint air layer that encloses all protrusions, whereas, in a cluster of cavities the emergence of unconnected air layers that are attached to a single cavity is to be expected. Clearly, in the second case, a localised disturbance will cause the breakdown of a few air layers close to the disturbance, while, in the case of a joint air layer, a much larger surface area will lose its air cover.

### Biomimetic realisation of the “ice cream cone” for blast furnace tuyères

#### Theoretical considerations

**Figure 5:**
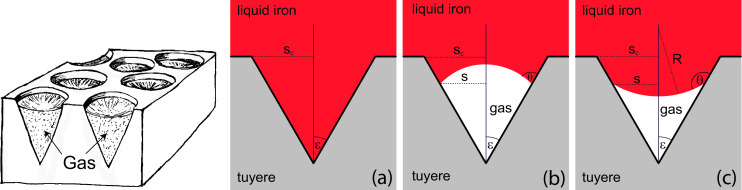
Left: Closely arranged “ice cream cones” on the surfaces of tuyères that contain gas pockets are able to mitigate heat inflow from an overflowing liquid iron sheet considerably (Drawing by Birgit Binder, Entringen. This content is not subject to CC BY 4.0). Right: The relation between the contact angle θ and the acute angle ε, which defines the cone geometry, is decisive for the existence and stability of the gas/liquid interfaces. Panels (a–c) show different possible states of the interface. (a) The cone is completely filled with liquid iron, and the system does not allow for the existence of a gas/liquid interface. (b) Contact angle and acute cone angle are related via θ *<* ε + 90°. A gas/liquid interface can exist, but it is neither stable, nor does it emerge by itself. Eventually, the iron will displace the air pocket, leading to the situation in (a). (c) Contact angle and acute cone angle satisfy the condition θ *>* ε + 90°, which guarantees existence and stability of the gas/liquid interface and of the air pocket it encloses. Figure 5 right (b) and (c) are from [47] and were reprinted by permission from Springer Nature from the journal Journal of Bionic Engineering (“When Lotus Leaves Prevent Metal from Melting – Biomimetic Surfaces for High Temperature Applications” by W. Konrad; J. Adam; S. Konietzko; C. Neinhuis), Copyright 2019 Springer Nature. This content is not subject to CC BY 4.0.

The great advantage of rotationally symmetric recesses (Figure 5) and the resulting, necessarily spherical interfaces [46] is their simple geometric structure, which can easily be described in terms of analytic functions.

When a sheet of liquid iron flows over the tuyère surface, a complex interaction between the gas in the recesses and the liquid evolves. It is, of course, hopeless to try to describe the interaction dynamics in terms of analytic functions. But once a more or less stationary state has been reached, which is usually the case when the cooling system of the tuyères becomes overstrained, simpler considerations are sufficient.

To discover whether the overflowing liquid iron wets the recesses completely, implying a massive heat inflow into the tuyère, or whether air pockets within the recesses survive, providing an efficient insulation against heat input, it is appropriate to compare the formation energy of the two possible states. The one with the lower energy demand will be realised, at least in the majority of indentations.

Calculation of the energy difference between the two situations (for details consult appendix D) results in a surprisingly simple relation: Leaving air pockets within the recesses is the energetically favourable option regarding the iron sheet if the contact angle θ and the acute angle ε of the cone satisfy the inequality


[3] θ>ε+90°.

Refer to Figure 5 for the definitions of θ and ε. If θ is given, the relation in Equation 3 restricts the acute cone angle to the interval


[4] 0<ε<θ−90°.

The next step is to clarify whether such air layers (or rather the interfaces that separate them from the liquid iron) do exist in the first place and whether they are stable against perturbations.

The existence problem (details are presented in appendix B) boils down to the question whether a cubic poynomial in *s* (*s* is the radius of the contact circle, see Equation 5 and Figure 5) has at least one real and positive solution. It can be shown that this is the case for all physically reasonable values of θ and ε. The mechanical stability of the interface is treated in appendix C. It turns out that the simple cone geometry (simple compared to the indentations shown in Figure 9) with a maximum radius *s*_c_ ≈ 5 mm is a good choice. Interfaces that evolve within it are stable, regardless of the values of θ and ε and the pressure of the liquid iron.

#### Successful test of the concept

Due to its excellent heat conduction, tuyères are manufactured from copper. Unfortunately, copper is readily wetted by liquid iron, the contact angle between liquid iron and copper amounts to θ ≈ 37°. Equation 3, however, requires clearly θ *>* 90°. The solution of this problem was to coat the copper surface with corundum (Al_2_O_3_), a material on which iron exhibits a contact angle of θ ≈ 130°. Equation 4 implies that the acute angle of the cone should then be in the interval 0 *<* ε *<* 40°.

**Figure 6:**
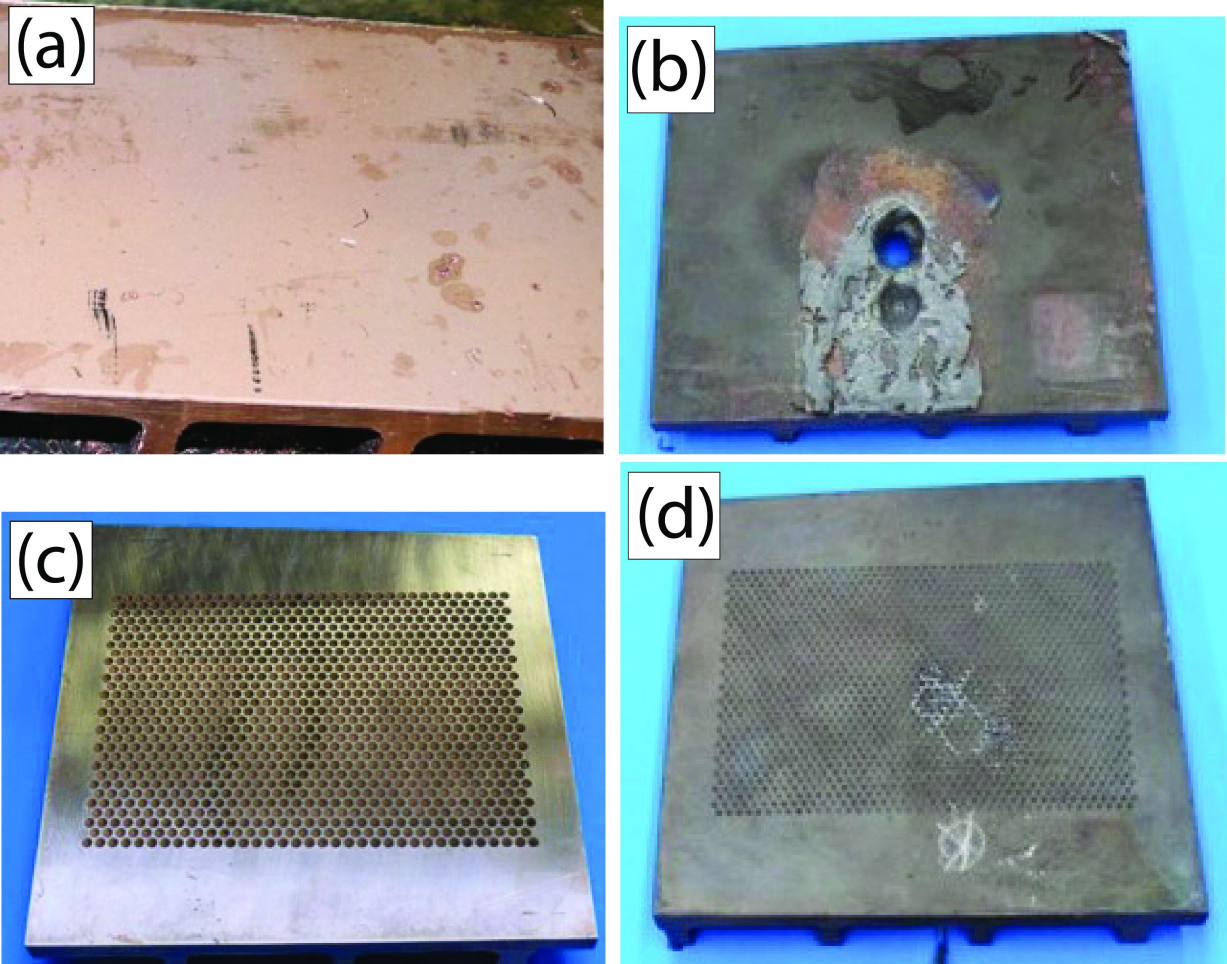
The two test objects before (a, c) and after (b, d) testing. (a, b) Unmodified copper plate, (c,d) copper plate equipped with cone-shaped cavities and a corundum coating. (Photos: Jörg Adam. Figure 6 is from [47] and was reprinted by permission from Springer Nature from the journal Journal of Bionic Engineering (“When Lotus Leaves Prevent Metal from Melting – Biomimetic Surfaces for High Temperature Applications” by W. Konrad; J. Adam; S. Konietzko; C. Neinhuis), Copyright 2019 Springer Nature. This content is not subject to CC BY 4.0.

To test our concept, a copper plate was furnished with hexagonally arranged cones (cone radius at the surface of the plate *s*_c_ = 2.5 mm, cone depth = 4.5 mm and acute angle of the cones ε ≈ 30°) and coated with corundum. Figure 6 shows the surface of the plate before and after 14 kg of liquid iron were poured over it within one minute. For comparison, an unmodified plate of pure copper was treated in a similar way (but merely with 5 kg of liquid iron, for details see [47]). As is obvious from Figure 6, the prototype showing the structured surface remained unharmed by the liquid iron.

### Conclusion: A broad understanding of biological function is beneficial for biomimetics

**Figure 7:**
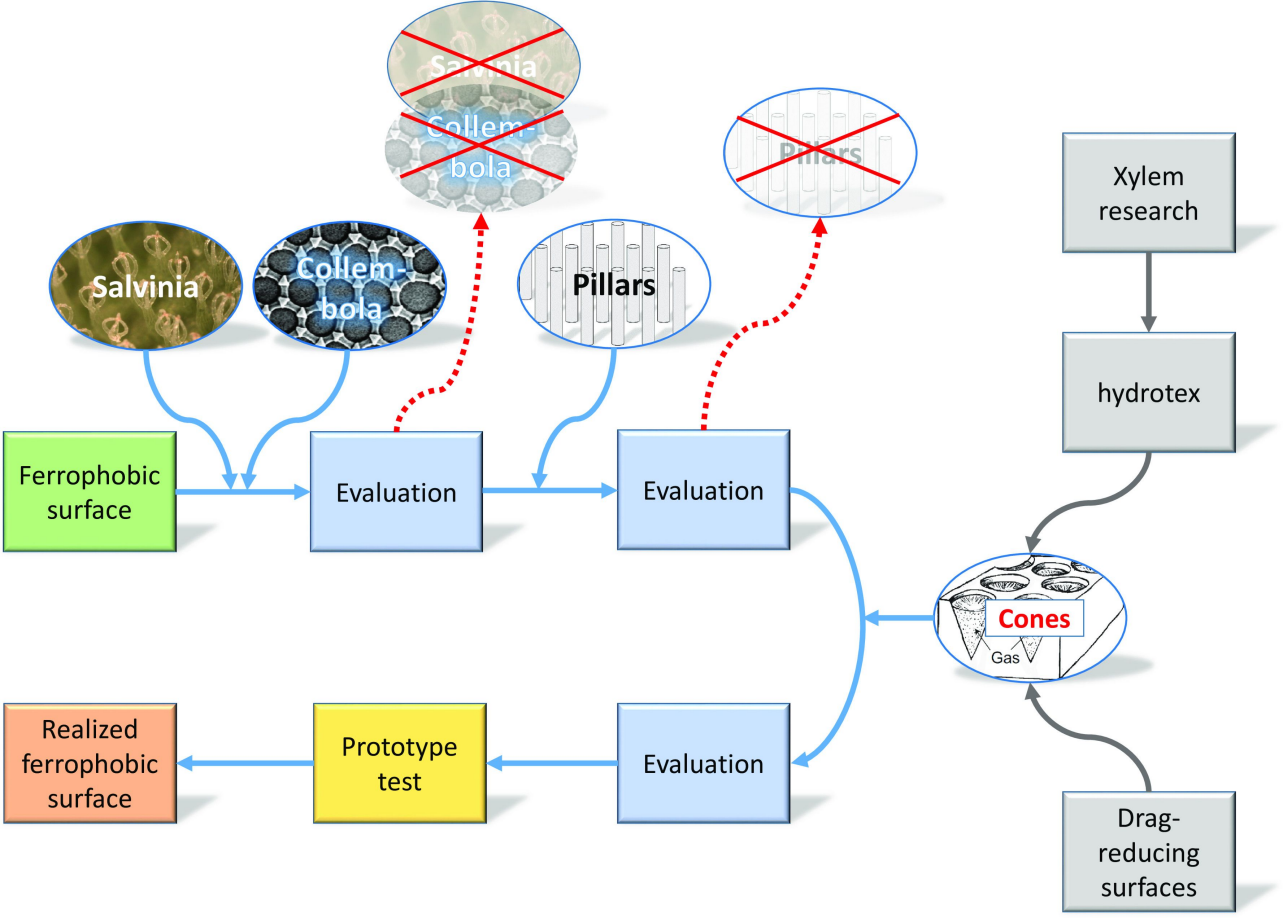
Sketch of the project progress. Initially, two biological models showing highly water-repellent surfaces, namely the arthropod group of Collembola and the water fern Salvinia molesta, were selected as biological models for the desired ferrophobic surface. These models, however, turned out to be not suitable; neither was a simplified design of rigid pillars (see text). In a next step, experiences from three former projects were considered, resulting in a surface decorated with cone-like indentations, which showed the desired liquid iron repellency. The projects are shortly described in appendix A.

The project history presented in this contribution illustrates that seemingly straight paths in biomimetics can turn out to be dead ends whereas following more complex approaches that delve much deeper into the biological complexities can be successful (Figure 7). We would like to emphasise that we do not intend to draw general conclusions from a single case study, even if it encompasses experience accumulated over a time span of 15 years. However, we feel entitled to draw the following conclusions.

One underestimated problem in biomimetics is that the biological function of a biological structure is quite often neither really clear nor well-defined [12,14,28]. Clarity and correctness of a function are, however, considered to be essential for identifying suitable biological models for biomimetic projects [7,11,15]. In fact, the central role of an identified biological function is at the heart of the top-down strategy and also the basis of facilitating biomimetic research by devising formal schemes of biomimetic work [15,48].

As described in the Introduction, biomimetic tools such as TRIZ or other databases structured according to biological functions seem to make the participation of biologists superfluous. A large collection of biological systems connected to defined functions would make biomimetic work possible without biological expertise [6–8]. This is an attractive prospect, as it promises uncomplicated road maps that can be followed by, for instance, engineers without complex interdisciplinary discussions. Most bioinspired designs to date followed, however, a bottom-up approach, meaning that they were initiated by a biological discovery and not by the top-down idea of tapping collections of models with biological functions [15].

The presented project example demonstrates the problem with the function in biomimetics quite nicely. The desired technical development was to devise an external surface of an object that repels a liquid, namely hot iron, reliably by allowing for the formation of an isolating stable air layer. It is then an obvious biomimetic routine to look for organisms showing external surfaces that repel water upon immersion with high efficacy.

Although the project was inspired by biological surfaces showing exactly this behaviour, *Salvinia molesta* and Collembola were not suitable for a technical transfer. The solution came from another biological structure with an apparently different and much more complex functional context, that is, the curved shape of micropores within walls of water-transporting conduits of plants. These structures might produce stable air layers to allow for refilling, which would mean a biological context of self-repair. It can be considered as quite impossible to make such complex functional contexts detectable by formal tools for users without a deeper understanding of biology.

By its core concept, biomimetics is an interdisciplinary endeavour, which attempts to bridge the gap between biology and engineering (or applied sciences in general). This gap is quite profound: Whereas biologists are interested in organisms, engineers are interested in designing successful technical constructions. Naturally, the aim of an applied science project (a biomimetic project is always an applied science project) is first and foremost the development of the desired technical item. This harbours the danger of considering the need to understand biology as a distraction rather than an asset (all the more so because real interdisciplinary cooperation can be strenuous due to differences in terminology, approaches and mentalities). In fact, there is increasing concern that an inadequate understanding of biology and insufficient evaluation of the richness of biological model systems and functional contexts will suppress the full potential of biomimetics [1,7,13,49]. The presented project example supports these claims.

## Appendices

### A. Related research projects into which one or more of the authors were involved

- “Drag-reducing air–water interfaces”, 2007–2010. Participants: University of Tübingen, University of Bonn, funded by the German Science Foundation (DFG), https://gepris.dfg.de/gepris/projekt/39399990/ergebnisse
- “Energy self-sufficient fibre based long-distance transport of liquids”, 2008–2011. Participants: University of Tübingen, Institut für Textil- und Verfahrenstechnik (Denkendorf), Helmholtz-Zentrum (Berlin), TWD Fibres GmbH (Deggendorf), funded by the German Federal Ministry of Education and Research (BMBF), http://edok01.tib.uni-hannover.de/edoks/e01fb13/742508609.pdf
- “Hydrotex”, 2012–2014. Participants: University of Tübingen, Institut für Marine Ressourcen (IMARE, Bremerhaven), Institut für Textil- und Verfahrenstechnik (Denkendorf), Frottana (Großschönau), Rivers and Tides Boatbuilding (Elsenfeld), funded by the German Federal Ministry for Economic Affairs and Energy (BMWi). Masters thesis generated from the project: Michael Münster, 2014, “Bionik wasserabweisender Oberflächen (Biomimetics of superhydrophobic surfaces)”, University of Tübingen, Department for Geosciences
- “Increase of energy efficiency of blast furnaces by using novel longlife-tuyères”, 2015–2018. Participants: Betriebsforschungsinstitut (Düsseldorf) (a research institute of the german steel industry), Hundt & Weber at Siegen (a german subsidiary company of Lebronce Alloys, a manufacturer of tuyères), Institute of Botany of the Technical University of Dresden, funded by the German Federal Ministry for Economic Affairs and Energy (BMWi)

### B. Existence of a gas/liquid interface and the Young–Laplace equation

#### General case

If the forces acting across an gas/liquid interface are in equilibrium, the Young–Laplace equation [50] states that the difference between liquid pressure *p*_l_ and gas pressure *p*_g_ is counterbalanced by the surface tension term 2σ*H*, according to


[1] $$p_{\text{g}}=p_{\text{l}}+2\sigma H.$$

σ denotes the surface tension and *H* the mean curvature of the gas/liquid interface. If *p*_l_ and *p*_g_ are constant along the interface, *H* is constant, too, and the interface belongs to the class of constant-mean-curvature surfaces, which are defined and studied in the field of differential geometry. This correspondence allows one to exploit and to apply purely mathematical results to practical problems. If the values of *p*_l_, *p*_g_ and *H* do not fulfill Equation 1 neither interface nor air layer form.

**Figure 8:**
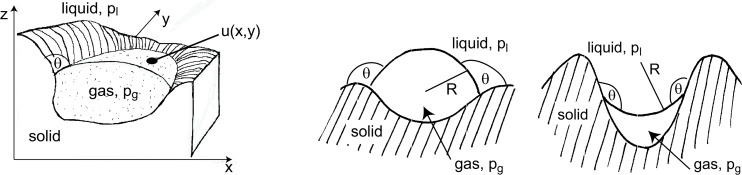
Illustration of the Young–Laplace equation. Left: The interface is given by the equation *z* = *u*(*x*,*y*). Centre and right: If the contact line exhibits rotational symmetry, the surface *z* = *u*(*x*,*y*) becomes a spherical section of radius *R*. The combination of the contact angle θ and the geometry of the solid dictate whether the gas/liquid interface is convex (*H* = 1/*R*) or concave (*H* = −1/*R*).

If Equation 1 is fulfilled by a given gas layer, the shape *z* = *u*(*x*,*y*) of the gas/liquid interface (see Figure 8) can be calculated as the solution of a differential equation. Expressing *H*, the mean curvature of the gas/liquid interface, as


[2] $$H=\frac{\left(1+u_{x}^{2}\right)u_{yy}-2u_{x}u_{y}u_{xy}+\left(1+u_{y}^{2}\right)u_{xx}}{2{\left(1+u_{x}^{2}+u_{y}^{2}\right)}^{3/2}}$$

(subscripts denote partial derivatives), the Young–Laplace equation (Equation 1) becomes a non-linear differential equation for the surface *u*(*x*,*y*). In order to obtain well-defined solutions suitable boundary values have to be prescribed, for example, the contact angle θ along the contact line where air, water and solid meet.

#### The case of rotational symmetry

Elementary geometry allows one to express the gas volume *V* shown in Figure 5 and the interface curvature *H* in terms of the cone geometry (*s*, θ and ε are defined in Figure 5) as


[6] $$V=\frac{\pi s^{3}}{3}\left(\cot\varepsilon +\frac{{\left[1-\sin\left(\theta -\varepsilon \right)\right]}^{2}\left[2+\sin\left(\theta -\varepsilon \right)\right]}{\cos^{3}\left(\varepsilon -\theta \right)}\right)$$

and


[7] $$H=\frac{\cos\left(\theta -\varepsilon \right)}{s}.$$

Insertion of Equation 1, Equation 6 and Equation 7 into the gas equation 
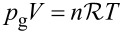
 (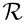: gas constant, *n*: number of gas particles, *T*: temperature) results in a cubic polynomial for the radius *s* of the contact line (see Figure 5):


[5]

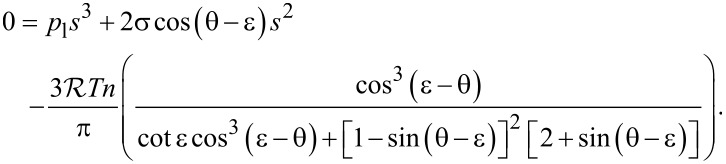



This polynomial has exactly one real and positive solution, provided *p*_l_ *>* 0, *T >* 0, 0 ≤ ε ≤ π/2 and 0 ≤ θ ≤ π. This implies that in situations compatible with these specifications an interface forms that has the shape of a spherical surface [46].

### C. Mechanical stability of a gas/liquid interface

An gas/liquid interface is mechanically stable if it reacts to a perturbation (such as a pressure fluctuation causing a small shift of the contact line) by returning back to its equilibrium position. (At the contact line, the gas/liquid interface touches the solid substrate.)

Upon Taylor expansion of the Young–Laplace equation and the gas equation 
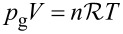
 around a point of mechanical equilibrium, it can be shown that the quantity


[8] $$\Psi ={\left[\frac{p_{\text{g}}}{V}+2\sigma \frac{\text{d}H}{\text{d}V}\right]}_{0}$$

indicates whether a mechanical equilibrium is stable (Ψ *>* 0) or not (Ψ *<* 0). *H* denotes the curvature of the interface (see appendix B) and the subscript 0 indicates that all quantities should be evaluated at the point of equilibrium [38–39,51–52].

**Figure 9:**
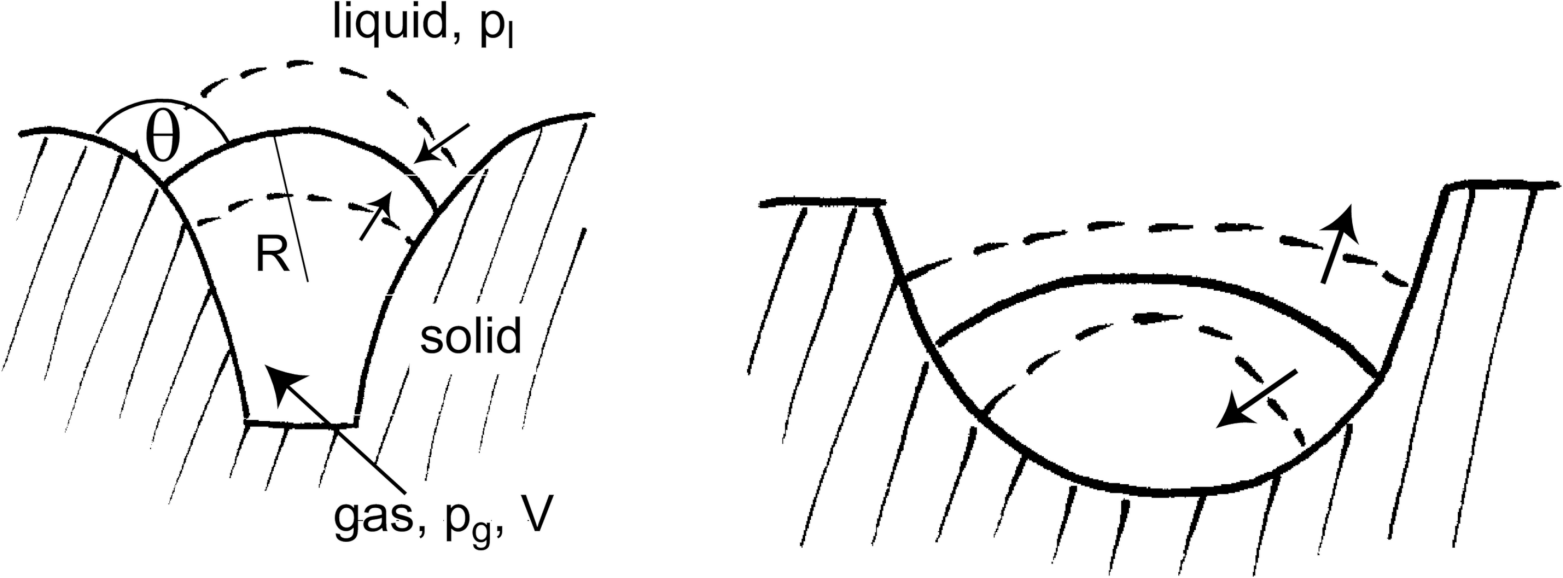
Mechanical stability of a gas/liquid interface. Left: After being deflected from its equilibrium position, the interface returns to the equilibrium position (Ψ *>* 0). Right: The interface reacts to a deflection by moving further away from the equilibrium position (Ψ *<* 0).

The term *p*_g_/*V* in Ψ is always positive, the sign of Ψ depends, therefore, on the behaviour of d*H*/d*V* when the gas volume is increased by the shift of the contact line: (i) If the interface curvature increases (left part of Figure 9), d*H*/d*V* and Ψ are positive, indicating a stable interface. (ii) If the curvature decreases, d*H*/d*V* is negative; if it exceeds *p*_g_/*V*, Ψ becomes negative, and the interface is unstable (right part of Figure 9).

If the indentation resembles an ice cream cone, such as in Figure 5, application of Equation 8 leads to Ψ *>* 0, regardless of the sign of *H* [47].

The surface tension in the iron/gas interface should also be able to restrain other forces that try to deform the interface (such as the gravitational force). This is guaranteed as long as the typical dimensions of a gas-holding structure (such as the maximum radius *s*_c_ of the contact circle) do not exceed the capillary length,


[9] $$l_{\text{c}}=\sqrt{\frac{\sigma }{\rho g}}\approx 4.8\text{ mm,}$$

which results from the surface tension σ ≈ 1.6 N/m and the density ρ ≈ 7000 kg/m^3^ of liquid iron and the gravitational acceleration *g* ≈ 9.81 m/s^2^ [50].

### D. Formation energy of a gas layer

When the gas pockets within cone-like structures on the tuyère surface are overflown by liquid iron they may stay fixed or they may become displaced. Both alternatives require the formation and/or destruction of surface and volume energy; it is reasonable to assume that the energetically favourable alternative will be realised. Comparison of Figure 5a with Figure 5b or Figure 5c allows one to calculate Δ*E*, the energy difference related to the alternatives:


[10] $$\Delta E=\Delta E_{\text{V}}+\Delta E_{\mathrm{lg}}+\Delta E_{\text{sg}}=\sigma \left[-2HV+A_{\mathrm{lg}}+A_{\text{sg}}\cos\theta \right].$$

Δ*E_V_*, Δ*E*_lg_ and Δ*E*_sg_ are the energies necessary to remove liquid iron of volume *V* (and to replace it by gas), to form a liquid/gas interface of area *A*_lg_ and to separate liquid iron from the solid across an area *A*_sg_, respectively (for details, see [47]).

Gas volume *V* and interface mean curvature *H* have already been calculated in Equation 6 and Equation 7 (see appendix B). Inserting them and the geometric relations *A*_lg_ = 2π*s*^2^/(1 + sin(θ − ε)) and *A*_sg_ = π*s*^2^/sinε (see Figure 5) into Equation 10 results in


[11]

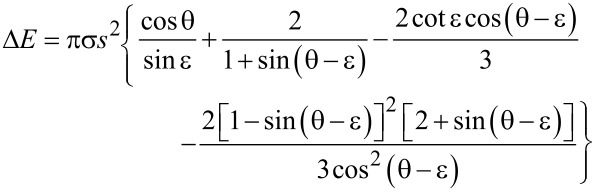



Obviously, the sign of Δ*E* depends only on the expression within the braces. Closer inspection shows that the energetically favourable case Δ*E <* 0 requires that the contact angle θ and the acute angle ε of the cone satisfy the inequality


[12]

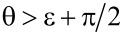



(a situation as depicted in the left part of Figure 5), whereas θ *<* ε + π/2 leads to Δ*E >* 0 (cf. right part of Figure 5).
